# The American Hospital Association

**Published:** 1907-10-26

**Authors:** 


### HOSPITAL ADMINISTRATION.

### CONSTRUCTION AND ECONOMICS.

### THE AMERICAN HOSPITAL ASSOCIATION.

The ninth annual conference of this Association has been recently held in Chicago, but a full report of the proceedings have not yet reached this country. It cannot fail to interest hospital workers throughout the British Empire to realise that this Association is a development of the Association of Hospital Superintendents, the object of which was to assemble annually those in immediate charge of hospitals for the interchange of ideas, comparing and contrasting methods of management, the discussion of hospital economics, the inspection of hospitals, the consideration and suggestion of better plans of operating them, and all other matters that might affect the general interests of the membership. Membership was active and honorary, the former being those who were at the time of their election the executive heads of hospitals, without reference to schools, and honorary membership was confined to members whose services public or private, entitled them to such recognition. The former pay five dollars a year subscription. Each annual conference was to be held at the place and on the dates fixed by the annual meeting or the executive committee of the Association. Special meetings for important business, to be set out in full, could, however, be held on the requisition of not less than five members, with the consent of the President, by giving at least sixty days' notice to every member of the Association. We have already recorded our admiration of the businesslike methods pursued at these annual conferences, and of the great value of many of the papers and much of the discussion of a practical character which have characterised their proceedings almost from the first. The originators of the Association of Hospital Superintendents in the United States may well be proud of the results which were attained by their Association in the first seven years.

#### Its Progressive Development.

How momentous for good the work of the Association was shown by the eighth conference held at Buffalo in 1906. Throughout the proceedings evidence was afforded of a desire to extend the influence of the Association by including within it all who took an active part in hospital affairs, and were really interested in the efficient management and maintenance of hospitals. This resulted in the name of the Association being then changed to the American Hospital Association, and in the addition of associate members, who are confined for the present to the officer next in authority below the superintendent. Such associate members are not entitled to vote. The meeting at Chicago this year was attended by some hundred and fifty members. Our correspondent informs us that "the first series of individual reports of the sub-committees of the Committee on Hospital Progress were very successful. This Committee has been increased from four to six members, and a further increase is contemplated next year. The plan is the same as that which is working so well in our local (*i.e.* New York) hospital conference—to cover gradually the whole field of hospital progress in a series of annual reports and studies, thus rounding out the work of each annual convention, and giving continuity and cumulative value to the work of the Association."

#### The Committee on Hospital Progress.

It may be well to explain that the Committee on Hospital Progress is charged with the duty of observing each year the development of hospital work in the United States and Canada, and of submitting a condensed report of its observations at the annual convention of the Association. The Committee on Hospital Progress is subdivided as follows: (*a*) a committee of one on hospital construction; (*b*) a committee of one on hospital efficiency, hospital finances, and the economics of administration; (*c*) a committee of one on medical organisation and medical education; (*d*) a committee of one on the training of nurses. It is further of interest to note that the reports and recommendations of this Committee are printed as part of the proceedings of the American Association. In this way the annual reports of the Association may become valuable, because they should contain precise information concerning the progress made each year in hospital equipment, ward organisation, medical organisation, nursing organisation, and business management. These are all questions of the deepest interest to everybody actively engaged in the administration of a hospital. The fact that in the United States they now have the special consideration of a permanent committee has resulted in expediting the development of hospitals upon right lines, for the Association at each annual convention is thus put in possession of everything that is worth knowing about the actual progress of hospitals throughout the country.

#### Local Hospital Conferences.

The Hospital Conference has been first established in New York, where forty institutions have joined it. It has gradually developed in its work as a permanent institution, and its main object is to seek the establishment of reciprocal relations between the hospitals themselves. It is wisely felt that the hospitals in every large city should be co-ordinate in their work in caring for emergency cases, in arranging for the adequate distribution of ambulance service, each of which represents a special problem, and demands reciprocal action by the hospitals in the interests not only of themselves but of the whole community.

### Hospital Trustees and Chairmen.

At Chicago another important amendment was made in the constitution of the Association, proving once more the extended interest in the work of hospitals which is resulting from the present movement. Hospital Trustees or Chairmen of Committees have now been made eligible for membership. It is hoped by this means to quadruple the numbers who will join the Association, and at the same time to acquire a fund in excess of the needs for the annual report and other charges. Any surplus, if it is created, is to be held available for all purposes of legitimate propaganda, and if its expenditure is wisely directed the welfare of the hospitals throughout the United States must be promoted. We are of opinion that the progress thus indicated and the objects aimed at should excite the interest of all hospital workers in this country, and lead, possibly, to the reconstitution of the Hospitals Association on this side. There can be no doubt that the year 1907-8 will be momentous for good in the history of the American Hospitals Association, for Dr. S. S. Goldwater, one of the most active of hospital superintendents, has been appointed president, and his colleagues include the superintendents of the Presbyterian Hospital, Chicago; the Hospital for Sick Children, Toronto; the Rhode Island Hospital, Providence; the Central Maine General Hospital, Lewiston; and the Grace Hospital, Detroit. The meetings of the Association are usually held about the third week in September, and we can confidently say that any English representative of a great hospital who has the time and opportunity to be present at one of these meetings is sure to gain much information of value, and to spend one of the pleasantest weeks he is likely to experience in the course of his life.

